# Concomitant treatment with sertraline and social skills training improves social skills acquisition in social anxiety disorder: A double-blind, randomized controlled trial

**DOI:** 10.1371/journal.pone.0205809

**Published:** 2018-10-29

**Authors:** Marcio Bernik, Fabio Corregiari, Mariangela Gentil Savoia, Tito Paes de Barros Neto, Cristiane Pinheiro, Francisco Lotufo Neto

**Affiliations:** Anxiety Disorders Program, Department and Institute of Psychiatry, University of Sao Paulo, Sao Paulo, Brazil; Brown University, UNITED STATES

## Abstract

**Objectives:**

To examine whether: (1) sertraline (SER) + psychotherapy is superior to psychotherapy alone; (2) group cognitive-behavioural therapy (GCBT) is superior to group psychodynamic therapy (GPT) and (3) SER+GCBT or SER+GPT is superior to Placebo (PLA)+GCBT or PLA+GPT in social anxiety disorder (SAD).

**Methods:**

A double-blind randomized controlled trial. Participants were assigned either to: SER+GCBT (n = 34); SER+GPT (n = 36); PLA+GCBT (n = 36) or PLA+GPT (n = 41) for 20 weeks. SER (or PLA) was administered at doses from 50 to 200 mg/d. Primary measures were both categorial: remission (CGI score≤2), response of social symptoms (≥50% reduction in Scale of Avoidance and Social Discomfort (SASD)); and continuous: reduction of SASD and Multidimensional Scale of Social Expression(M-MSSE).

**Results:**

SER exhibited better improvement of social anxiety symptoms rate than PLA (25.73% *vs*. 9.46%, *P* < .05). Neither GCBT differed from GPT (12.33% *vs*. 22.54%, *P* = .11) nor SER+GCBT from PLA+GCBT (17.65% vs. 7.69%, *P* = .20). However, SER+GPT was superior to PLA+GPT (33.33%, vs. 11.43%, *P* < .05). M-MSSE had superior improvement for SER+GCBT vs PLA+GCBT (*P* < .01) but not for SER+GPT vs. PLA+GPT (*P* = .80). SASD scores improvement were greater for SER than PLA (*P* < .01) and for SER+GCBT vs. PLA+GCBT (*P* < .05), but neither GCBT differed from GPT(*P* = .60) nor SER+GPT differed from PLA+GPT (*P* = .09).

**Conclusions:**

In overall, SER+psychotherapy was superior to psychotherapy alone. SER potentiated GCBT by enhancing social skills acquisition.

**Trial registration:**

ISRCTN 57551461.

## Introduction

Psychotherapy, pharmacotherapy or both are the fundamentals of social anxiety disorder (SAD) treatment. Among medications, selective serotonin reuptake inhibitors (SSRI) are the most extensively studied (for a review see [1]). There is also evidence, including qualitative and meta-analytic reviews, that cognitive-behavioural therapy (CBT) is beneficial for patients with SAD (e.g., [2]; [3]; [4]; [5], [6]). Other psychotherapy formats (e.g. psychodynamic) are also frequently used in SAD [7], [8], but efficacy evidence is smaller [9], [10], [11]. On the other hand, data from both psychotherapy and pharmacotherapy studies show that, although active treatments are statistically superior to control conditions, a substantial numbers of patients fail to complete treatment or achieve remission or clinically significant improvement [12]**.**

Regarding CBT, Ponniah and Hollon [5] had reported that social skills training (SST) alone is not efficacious for improving social skills in adult SAD. This conclusion is consistent with the accepted practice that exposure therapy is an essential component of treatment for phobic avoidance disorders [13]. Therefore, experiencing and mastering aversion during exposure procedures is, putatively, a core element of CBT effectiveness [14].

Serotonin (5-HT) plays a major role in central nervous system (CNS) structures underlying stress endurance [15]). Selective serotonin reuptake inhibitors (SSRIs) increase bioavailability of 5-HT in these brain structures (for a review see [16]). Thus , combining serotonergic drugs with CBT protocols incorporating exposure therapy (an anxiety eliciting therapy) would putatively result in synergist effects. For therapies not eliciting anxiety, there is no clear prediction for this interaction.

Accordingly, several SAD treatment guidelines recommend some combination of medication and psychological treatment [17]; [18]. On the other hand, a meta-analysis [19] found scarce evidences regarding advantages in combined therapies. For the pooled significant response of social symptoms rate, there was only a trend towards combined medication-psychological treatments over psychological treatment alone. Most were small studies sponsored by universities and public sources. Thus, they may not have been adequately powered [19]. To our knowledge, no other studies have been published comparing the efficacy of a SSRI plus more than one psychological treatment. Thus, further studies comparing efficacy of CBT, non-CBT therapy and combined medication and psychotherapy (with different methodologies) for SAD are still necessary.

Given the scarcity of evidence, the goals of this study were to examine: (1) whether pharmacotherapy with an SSRI and psychotherapy would be superior to psychotherapy alone in the treatment of SAD patients; (2) whether group cognitive-behavioural therapy (GCBT) would be superior to group psychodynamic therapy (GPT) and (3) whether GCBT or GPT are different regarding possible additive or synergistic effects with a SSRI (sertraline).

## Methods

### Participants

Recruitment occurred from April 5th 1999 through Jan 17th 2002. The follow-up period took place from May 25th 1999 to Nov 7th 2002 at the Anxiety Disorders Program of the Institute of Psychiatry, Sao Paulo University Medical School. Recruitment ended when planned number of individuals was reached. This clinical trial was not registered prior to patients’ enrolment as this was not the practice at that time. All ongoing and related trials for this drug/intervention are now registered. Given the absence of published similar trials at the time, sample size was calculated considering an alpha of 0.05 (2-tailed), a remission rate (defined as described below) of 15% in the comparator group and 45% in the sertraline group for a power of 80%.

Participants, aged from 18 to 65 years, were interviewed first by a clinical psychologist using an specific protocol interview and then by a psychiatrist using the Structured Clinical Interview for DSM-IV [20]. Subjects had to meet criteria for SAD diagnosis, with or without comorbid depression. Symptoms duration should exceed one year. Exclusion criteria included: clinically significant suicidal risk, Beck Depression Inventory (BDI [21]) score greater than or equal to 30; Hamilton Depression Rating Scale (HAMD [22]) score greater than or equal to 21; any other major psychiatric DSM-IV diagnosis, any medical-systemic disease possibly affecting mental condition including epilepsy; intake of more than two units of alcohol/day and/or current use of antidepressant medications or benzodiazepines. Risk of suicide was assessed by considering the corresponding items from the HAMD and BDI and by clinical judgment.

The hospital ethics committee (CAPPesq—Ethics Committee for Analysis of Research Projects at Clinics Hospital, Faculty of Medicine, University of São Paulo) approved the protocol. Study personnel explained the project verbally to each participant. All participants signed informed consent forms.

Among 178 potential participants, 146 (82%) were selected and randomly assigned to one of the four parallel treatment groups through a 1:1:1:1 randomization schedule (using a programmed Excel Microsoft Office spreadsheet). A research assistant (CP) not involved in other steps of the study performed the assignment and kept each patient group assignment in a sealed envelope.

**Fig 1:**
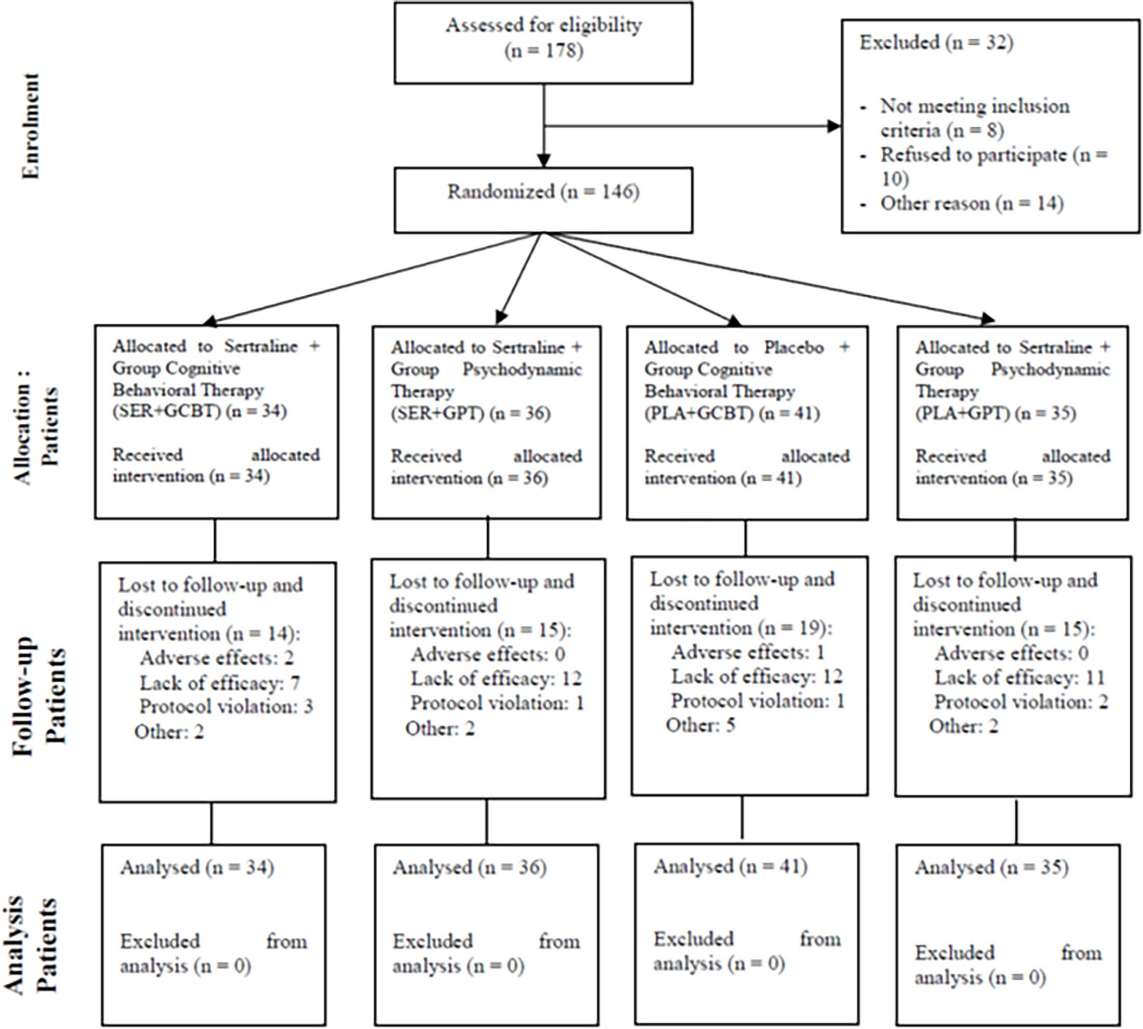
Consort flow chart.

The four treatment conditions were: sertraline + group cognitive-behavioural therapy (SER+GCBT, n = 34); sertraline + group psychodynamic therapy (SER+GPT, n = 36); placebo + GCBT (PLA+GCBT, n = 41) and placebo + group psychodynamic therapy (PLA+GPT, n = 35) (Fig 1).

### Outcome measures

The categorical primary outcome measure for symptom improvement were (1) remission rate and (2) significant response of social symptoms rate at the 20^th^ week. Remission rate was defined as a score of 1 (very much improved) or 2 (much improved) in the Clinical Global Impression-Improvement scale [23]. Significant response of social symptoms was defined as more than 50% reduction in the Scale of Avoidance and Distress Scale (SADS) [24].

Because SAD affects social and vocational behaviours, we emphasized both symptom reduction and behavioural modification as primary efficacy measures. Thus, as primary continuous efficacy measure were time-by-treatment interaction of the SADS score and the score at Multidimensional Scale of Social Expression–Motor Part (M-MSSE, [25]). The M-MSSE consists in a 64-item questionnaire developed to evaluate the expression of skilful social behaviours.

Secondary efficacy measures included:

- Final score in M-MSSE;
- Final score in SADS;
- Scale of fear of negative evaluation (FNE, [26]) final score and time-by-treatment interaction;
- Clinical Global Impression—severity (CGI–S) final score and time-by-treatment interaction;
- Clinical Global Impression–Improvement (CGI–I) final score
- Hamilton Anxiety Rating Scale (HAM-A; [27]) final score and time-by-treatment interaction;
- Hamilton Depression Rating Scale (HAM-D;) final score and time-by-treatment interaction;
- Beck Depression Inventory (BDI) final score and time-by-treatment interaction;

Safety assessments were based on SAFTEE questionnaire [28] and were assessed at the basal visit and at every visit thereafter.

### Design

**Fig 2:**
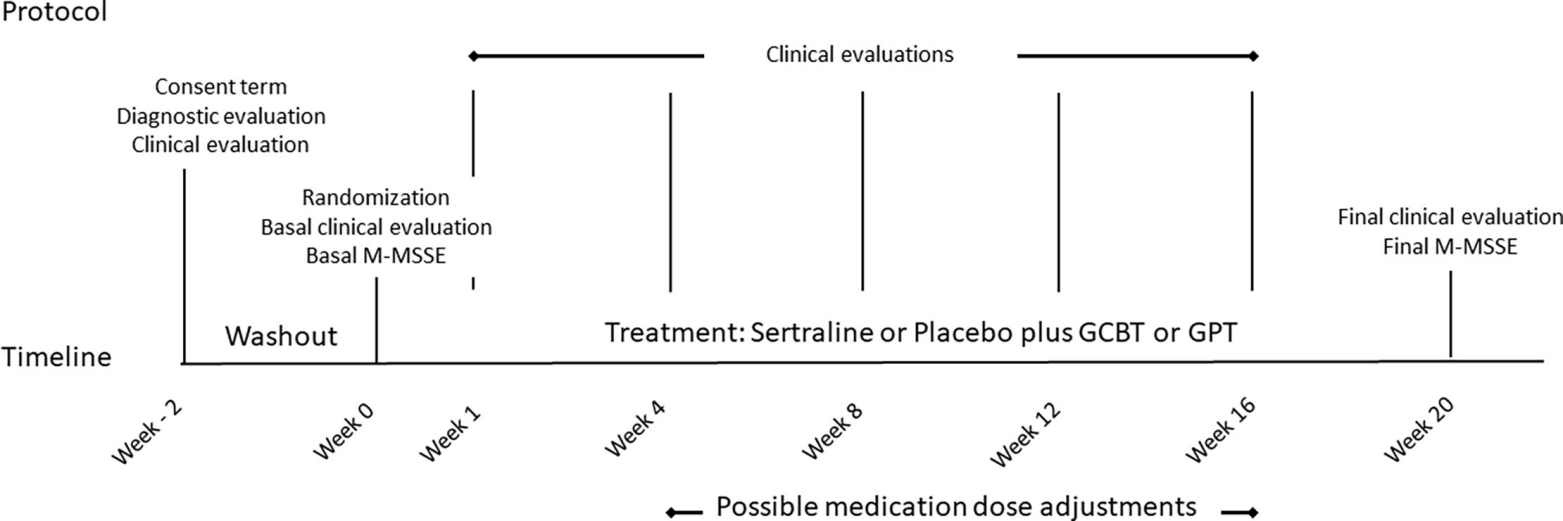
Study design diagram. M-MSSE: Multidimensional Scale of Social Expression–Motor Part; GCBT: Group Cognitive Behavioral Therapy; GPT: Group Psychodynamic Therapy.

Possible participants/individuals were initially assessed two weeks prior to randomization (week -2). In this occasion, the consent term was signed, the diagnostic evaluation was performed. Possible participants/individuals were also checked for inclusion and exclusion criteria. Between week -2 and week 0, all patients received identical placebo pills (washout period). After randomization at week 0, treatment lasted 20 weeks and patients were evaluated at weeks 0, 1, 4, 8, 12, 16, and 20 (Fig 2).

All patients received identical pills throughout the study. Psychiatrists, therapists and patients were unaware of the patient's treatment group allocation.

### Treatments

Patients were randomly assigned to receive group cognitive behavioural therapy (GCBT) or group psychodynamic therapy (GPT) and sertraline or pill placebo. Thus, there were four possible treatment conditions: sertraline+GCBT (SER+GCBT group); sertraline+GPT (SER+GPT group); placebo+GCBT (PLA+GCBT group); placebo+GPT (PLA+GPT group).

Therapists provided treatment in GCBT and GPT following: a standardized treatment manual for GCBT and more psychodynamic instructions for GPT. GCBT involved twenty 90-minute sessions. Subjects assigned to GCBT received training in anxiety-management skills and social skills followed by behavioural exposure to anxiety-provoking situations. GPT involved twenty 90-minute sessions of psychodynamic psychotherapy without training in anxiety-management skills, social skills or behavioural exposure. All therapy sessions were recorded and 30% were randomly selected and analysed by independent raters for adherence to the psychotherapy manual. All Therapist providing GCBT were experienced behaviour therapists and therapists providing GPT were also experienced therapists of mostly psychodynamic background.

Follow up medical / pharmacotherapy consultations involved eight sessions of 45 minutes each that included reviews and ratings of the severity of subjects’ anxiety, their response to treatment, and adverse events. Sertraline or pill placebo were administered on a fixed-flexible schedule beginning with 50 mg per day for sertraline and adjusted up to 200 mg per day. At week 4 and later, those individuals considered mildly ill or worse and with minimal side effects were eligible for dose increments. If BDI score exceeded 30 points at any time of the study or HAMD score exceeded 21 points, the case was discussed with the the team leader to evaluate subject's exclusion from the protocol. Nevertheless, no one was excluded under this condition.

### Adverse events

Symptoms possibly attributable to treatment were evaluated by means of the SAFTEE Scale, which was applied to the participants each visit, regardless of treatment assignment.

### Statistical analyses

Participant’s ' last observation carried forward data (LOCF) was used for data analysis. To be included in the intent-to-treat sample, individuals needed to have at least one efficacy reassessment after baseline.

Data were analysed using SPSS (IBM Corp. Released 2013. IBM SPSS Statistics for Windows, Version 22.0. Armonk, NY: IBM Corp.). Kolmogorov-Smirnov test and Levene’s test were used to evaluate normality of distribution and homogeneity of variances, respectively, prior to any statistical testing.

Independent samples *t*-tests were used to examine differences between groups. Mann-Whitney U Test was used to examine variables that did not follow a normal distribution. Categorical data were compared using Chi-square test and Fisher’s exact test.

General linear Models were used to examine changes over time in treatment groups. IBM SPSS Generalized Linear Models relaxes the assumption of normality for the error term and requires only that the dependent variable be linearly related to the predictors through a transformation or link function. Pairwise comparisons were planned a priori, namely, PLA *vs*. SER; GCBT *vs*. GPT; SER+GCBT *vs*. PLA+GCBT; SER+GPT *vs*. PLA+GPT.

Alpha was set at 0.05 in all analyses, without adjustment for multiple comparisons.

## Results

### Demographic and clinical characteristics

There were no baseline demographic characteristics differences between study groups (Table 1). The average age of participants was 33.8±9.5 years (range: 19–58 years). There were 82 males (56.2%) and 64 females (43.8%). Most of them were white (70.0%), single (60.1%), and had at least 12 years of education (78.5%). At baseline, subjects had moderate-to-severe anxiety symptoms and mild depression (Table 2). Some between group baseline differences were nevertheless observed in some clinical variables (Table 2). HAMA and HAMD were different in the overall comparison (χ^2^ = 8.80; *df* = 3; *P* = 0.032 and χ^2^ = 15.23; *df* = 3; *P* = 0.002 respectively). In the pairwise analyses, PLA+GPT had shown less anxiety symptoms than SER+GCBT and PLA+GCBT groups and less depression than all other groups (all *P*<0.05). The mean CGI Severity subscale rating was 4.65±1.71 (markedly ill) for all patients prior to treatment.

**Table 1:** Baseline sociodemographic characteristics of the subjects.

		SER+GCBT (n = 34)	SER+GPT (n = 36)	PLA+GCBT (n = 41)	PLA+GPT (n = 35)	All subjects (n = 146)	*P*
**Age (yrs.; mean±SD)**	** **	32.97±8.14	34.81±9.82	35.15±10.97	31.83±8.64	33.76±9.53	0.50^1^
**Female/Male** **sex—nº (%)**	** **	16/18 (47.06/52.94)	18/18 (50.00/50.00)	18/23 (43.90/56.10)	12/23 (34.29/65.71)	64/82 (43.84/56.16)	0.57^2^
**Race—nº (%)**	** **						0.09^3^
** **	**White**	24 (70.59)	26 (72.22)	26 (63.41)	26 (74.29)	102 (69.86)	
** **	**Black**	4 (11.76)	3 (8.33)	1 (2.44)	1 (2.86)	9 (6.16)	
** **	**Predominantly** **white/black**	3 (8.82)	7 (19.44)	5 (12.20)	7 (20.00)	22 (15.07)	
** **	**Asian**	2 (5.88)	0 (0.00)	6 (14.63)	0 (0.00)	8 (5.48)	
** **	**Other**	1 (2.94)	0 (0.00)	3 (7.32)	1 (2.86)	5 (3.42)	
**Level of** **Education**	** **						0.98^3^
** **	**Incomplete Elementary School**	2 (5.89)	2 (5.56)	2 (5.88)	2 (5.71)	8 (5.56)	
** **	**Elementary School**	5 (14.71)	5 (13.89)	7 (13.89)	6 (17.14)	23 (15.97)	
** **	**High school**	16 (47.01)	19 (52.78)	22 (56.41)	17 (48.57)	74 (51.39)	
** **	**College**	11 (32.4)	10 (30.56)	8 (10.51)	10 (28.57)	39 (27.08)	
**Marital Status (%)**	** **						0.89^3^
** **	**Married**	11 (33.33)	13 (36.11)	12 (30.77)	9 (25.71)	45 (31.47)	
** **	**Single**	21 (63.64)	19 (52.78)	23 (58.97)	23 (65.71)	86 (60.14)	
** **	**Widow/Divorced** **/Separated**	1 (3.03)	4 (11.11)	4 (10.26)	3 (8.57)	12 (8.39)	

^1^Kruskal-Wallis test

^2^Pearson chi-square

^3^Fisher exact test

**Table 2:** Description of the evolution of symptoms in the four groups in weeks 0 and 20.

	SER+GCBT (N = 34 for all measures except M-MSSE)	SER+GPT (N = 36 for all measures except M-MSSE)	PLA+GCBT (N = 41 for all measures except M-MSSE)	PLA+GPT (N = 35 for all measures except M-MSSE)	*P*-Value for overall comparisons between groups
**BDI-0**	17.65±7.89	15.11±10.21	15.05±9.86	11.97±7.35	0.08^1^
**BDI-20**	9.15±9.07***	9.36±8.86***	10.02±7.72***	10.29±8.68	0.87^2^
**HAMD-0**	**12.24±5.13**^**c**^	**10.69±6.63**^**a**^	**10.51±5.00**^**b**^	**7.63±3.75**^**c**^^**,**^ ^**a**^^**,**^ ^**b**^	<**0.01**^2^
**HAMD-20**	6.35±4.62***	6.08±5.73***	6.41±4.66***	4.74±3.42***	0.37^2^
**HAMA-0**	**15.18±5.56**^**b**^	14.28±7.41	**14.17±5.94**^**a**^	**11.60±3.70**^**b**^^**,**^ ^**a**^	<**0.05**^2^
**HAMA-20**	7.53±4.85***	7.53±6.14***	9.15±6.11***	7.80±4.18***	0.45^2^
**CGI-S-0**	4.53±0.66	4.56±0.69	4.66±0.73	4.37±0.60	0.25^2^
**CGI-S-20**	**3.35±1.30**^a^***	3.42±1.25***	3.88±1.27***	**3.97±1.10**^a^*	0.08^2^
**SADS-0**	23.76±4.06	21.69±6.71	22.22±8.15	21.34±6.41	0.23^2^
**SADS-20**	17.50±7.75***	16.58±9.91***	19.24±8.55**	18.71±8.22**	0.63^2^
**FNE-0**	25.06±4.80	24.86±4.41	24.24±5.66	24.00±5.42	0.89^2^
**FNE-20**	21.12±6.89**	21.44±7.54**	21.59±7.35***	23.11±6.07	0.63^2^
**M-MSSE-0**	148.09±9.15 (N = 25)	137.14±17.25 (N = 29)	134.50±28.76 (N = 28)	143.33±22.49 (N = 23)	0.07^2^
**M-MSSE-20**	127.56±11.43** (N = 11)	138.44±25.25 (N = 14)	129.48±20.78* (N = 16)	137.84±17.32 (N = 12)	0.22^2^

Mean±Standard Deviation

*P<0.05

**P<0.01

***P<0.001 for week 0 vs 20 comparison (*T*-test or Wilcoxon signed-rank test as appropriated)

a. P<0.05

b. P<0.01

*c*. *P<0*.*001 between assigned groups* (Mann-Whitney test)

SER = Sertraline; PLA = Placebo; GCBT = group cognitive-behavioural therapy; GPT = group psychodynamic therapy; BDI = Beck Depression Inventory; FNE = Scale of fear of negative evaluation; CGI-S = Clinical Global Impression–severity; HAMA = Hamilton Anxiety Rating Scale; HAMD = Hamilton Depression Rating Scale; SADS = Scale of Avoidance and Distress Scale; M-MSSE = Multidimensional Scale of Social Expression–Motor Part. 0 = week 0; 20 = week 20.

^1^ANOVA

^2^Kruskal-Wallis test

### Patient attrition rates

Rates of discontinuation were 32.4% (11/34) in the SER+GCBT group, 41.7% (15/36) in the SER+GPT group, 43.9% (18/41) in the PLA+GCBT group and 42.9% (15/35) in the PLA+GPT group (Table 3). Those rates were not different when examining all groups jointly (χ2 = 1.24, df = 3, p = 0.75).

**Table 3:** Number of dropouts per group at each study visit.

Week	1	4	8	12	16	20	Total
**SER+GCBT**	3	1	3	2	0	2	11
**SER+GPT**	2	4	1	1	7	0	15
**PLA+GCBT**	7	4	3	2	1	1	18
**PLA+GPT**	0	4	6	3	2	0	15

SER = Sertraline; PLA = Placebo; GCBT = group cognitive-behavioural therapy; GPT = group psychodynamic therapy.

### Efficacy results

#### Categorical primary efficacy outcomes

44.5% (65/146) of patients achieved remission, i.e. were rated as very much or much improved in the Clinician Global Impression-Improvement scale. Patients receiving sertraline did not have a greater probability of remission than those on placebo [35/70 (50.0%) *vs*. 30/76 (39.5%), Pearson χ2 = 1.64; df = 1; *P* = 0.20; OR = 1.53, 95% CI: 0.80–2.96].

Patients allocated to receive GCBT did not differ from those who had received GPT [36/75 (48.0%) *vs*. 29/71 (40.8%), Pearson χ2 = 0.76; df = 1; *P* = 0.39; OR = 1.34, 95% CI: 0.69–2.57]. There were also no differences in the probability of remission between SER+GCBT *vs*. PLA+GCBT [18/34 (52.9%) *vs*. 18/34 (43.9%) respectively, Pearson χ^2^ = 0.61; *df* = 1; *P* = 0.49; OR = 1.44, 95% CI: 0.58–3.58], and between SER+GPT *vs*. PLA+GPT [17/36 (47.2%), *vs*. 12/35 (34.3%), χ^2^ = 1.23; *df* = 1; *P* = 0.34; OR = 1.72, 95% CI: 0.66–4.46] at week 20.

Twenty-five from 144 (17.4%) patients had achieved clinically significant response of social anxiety symptoms, i.e. those with more than 50% reduction in the SADS Scale. Patients allocated to receive sertraline had a greater probability of significant response of social symptoms versus placebo [18/70 (25.7%) *vs*. 7/74 (9.5%), Pearson χ^2^ = 6.63; *df* = 1; *P* = 0.01; OR = 3.31, 95% CI: 1.29–8.53]. Those allocated to receive GCBT did not differ from those who had received GPT [9/73 (12.3%) *vs*. 16/71 (22.5%), Pearson χ^2^ = 2.61; *df* = 1; *P =* 0.11; OR = 0.48, 95% CI: 0.20–1.18). There were no differences in the probability of significant response of social symptoms between SER+GCBT *vs*. PLA+GCBT [6/34 (17.6%) *vs*. 3/39 (7.7%), Pearson χ^2^ = 1.67; *df* = 1; *P* = 0.29 (Fisher exact test); OR = 2.57; 95% CI 0.59–11.20), but SER+GPT was superior to PLA+GPT [12/36 (33.3%), *vs*. 4/35 (11.4%), Pearson χ^2^ = 4.88; *df* = 1; *P*<0.05; OR = 3.88; 95% CI: 1.10–13.53).

#### Continuous efficacy outcomes

There was clinical improvement in all primary and secondary continuous efficacy measures from week 0 to week 20 in the four groups except for BDI, FNE and M-MSSE in PLA+GPT group and M-MSSE in PLA+GCBT group, in which no improvement was observed. Mean scores, score changes and standard deviations for all primary and secondary continuous measures at endpoint (week 20) are presented in Table 2.

#### Primary continuous efficacy measures

**Fig 3:**
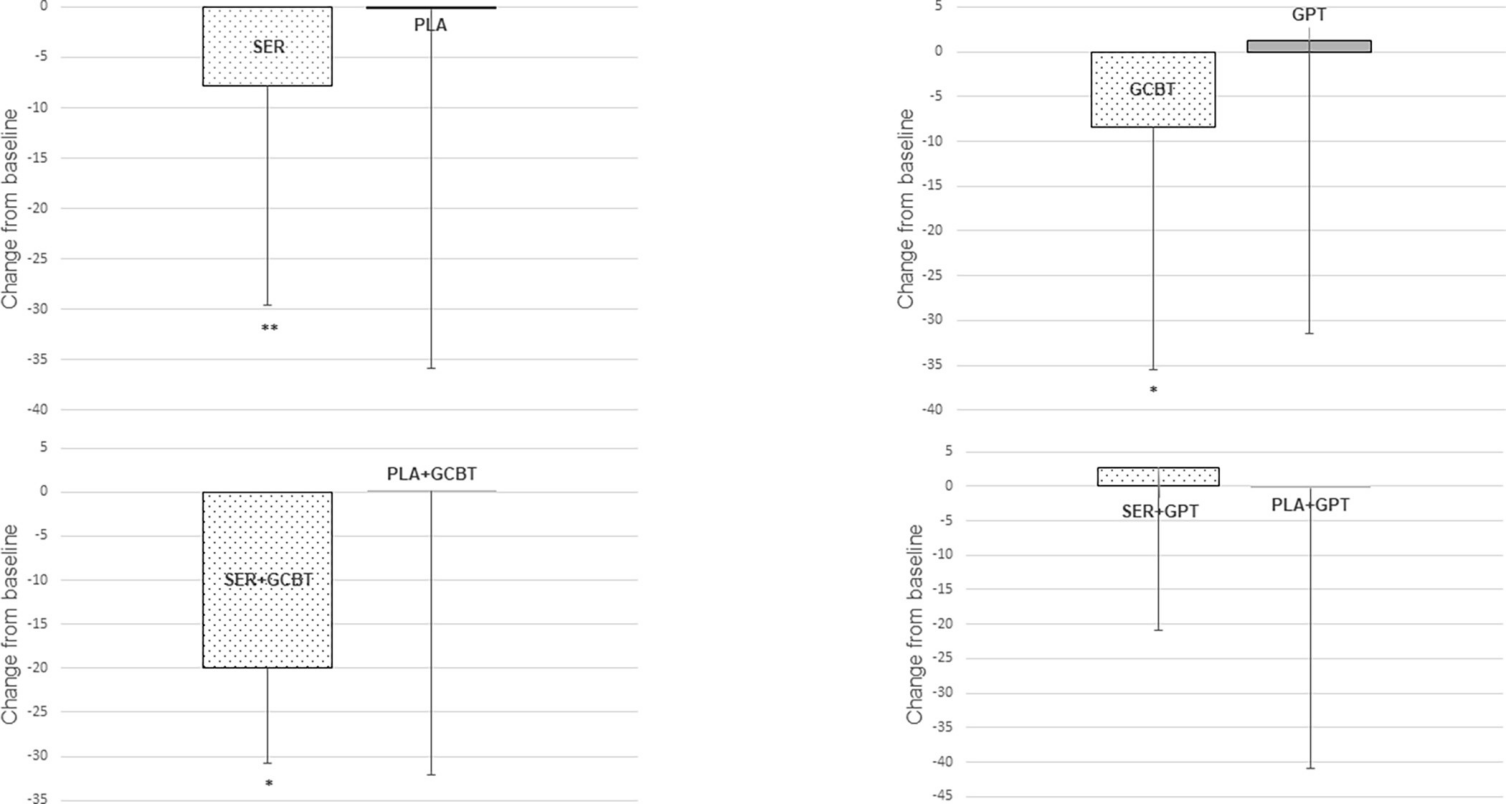
Mean change from baseline to week 20 in Multidimensional Scale of Social Expression–Motor Part (M-MSSE) score; SER = Sertraline; PLA = Pill Placebo; GCBT = Group Cognitive-Behavioral Therapy; GPT = Group Psychodynamic Therapy; *P<0.05 **P<0.01. Error bars represent one standard deviation.

There were no differences in final M-MSSE scores between sertraline and placebo (Final Estimated Marginal Means (FEMM): 133.53±20.50 *vs*. 132.97±20.48 *Z* = -0.78; *P* = 0.44), and there was a trend for GCBT superiority over GPT (FEMM: 128.93±19.70 *vs*. 137.66±20.90 *Z* = -1923; *P =* 0.054; Fig 3). In the general linear model, time-by-treatment interaction was not different between sertraline and placebo (FEMM: 133.53±20.47 *vs*. 132.97±20.48; *Z* = 0.38; *P* = 0.54; Effect Size (ES): η^2^p = 0.006, 95% CI: 0–0.092). GCBT had shown superior improvement over GST (FEMM: 128.94±19.99 *vs*. 137.66±19.99; *Z =* 4.87; *P* = 0.031; ES: η^2^p = 0.072, 95% CI: 0–0.21). Pairwise comparisons revealed a superior improvement in time-by-treatment interaction of M-MSSE score at week 20 for SER+GCBT group in comparison with PLA+GCBT (FEMM: 127.93±18.22 *vs*. 129.68±18.20; *Z* = 8.88; P<0.01; ES: η^2^p = 0.223, 95% CI: 0.02–0.44) and no difference between SER+GST *vs*. PLA+GST (FEMM: 138.44±22.25 *vs*. 136.87±22.25; *Z* = 0.065; *P* = 0.80; ES: η^2^p = 0.002, 95% CI: 0–0.117; Fig 3).

**Fig 4:**
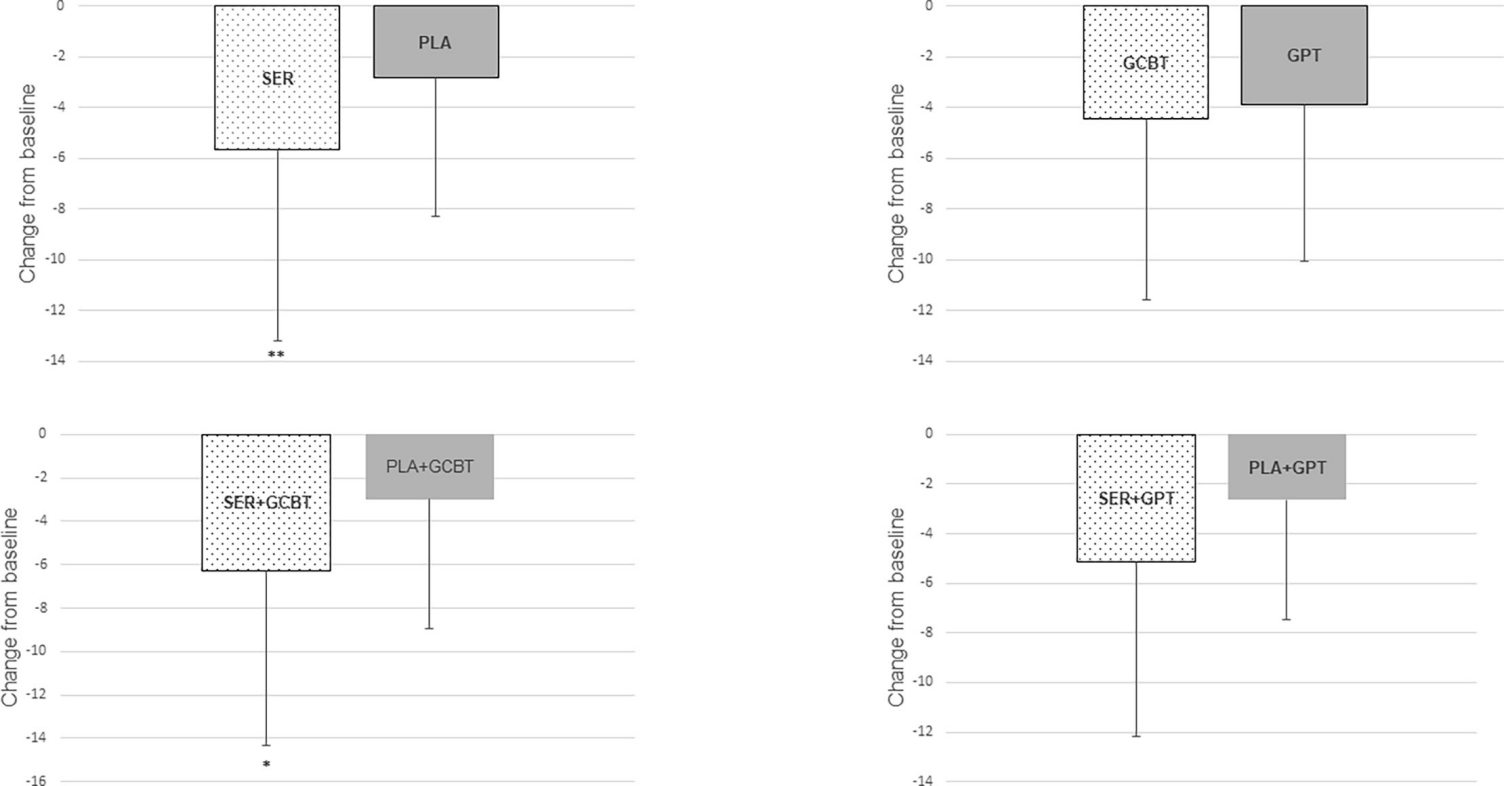
Mean change from baseline to week 20 in Scale of Avoidance and Social Discomfort (SASD) score (last observation carried forward); SER = Sertraline; PLA = Pill Placebo; GCBT = Group Cognitive-Behavioral Therapy; GST = Group Support Therapy; *P<0.05 **P<0.01. Error bars represent one standard deviation.

There was a significant reduction in SADS scores at week 20, both in patients receiving sertraline (P<0.001) and placebo (P<0.001). The general linear model revealed a time-by-treatment interaction with sertraline being superior to placebo in this reduction (FEMM: 17.03±8.60 *vs*. 19.00±8,60; *Z* = 6.96; *P* = 0.009; ES: η^2^p = 0.046, 95% CI: 0.00–0.13; Fig 4).

Patients randomized to GCBT and to GPT also presented a reduction in SADS from week 0 to 20 (Z = -4.96; Z = -4.61, respectively; both *P*<0.001) but the general linear model did not show a time-by-treatment (GCBT or GPT) interaction (FEMM: 18.45±8.65 *vs*. 17.63±8.65; *Z = 0*.*274; P* = 0.60; ES: η^2^p = 0.002, 95% CI: 0.00–0.04; Fig 4).

All four combined treatment groups showed improvement in SADS from week 0 to 20 (all *P*<0.001). Pairwise comparisons revealed a greater reduction of SADS score at week 20 for SER+GCBT group in comparison with PLA+GCBT (17.50±8.92 *vs*. 19.24±8.20; Z = 4.12; *P*<0.05; ES: η^2^p = 0.053, 95% CI: 0.00–0.18; Fig 4), but not for SER+GPT versus PLA+GPT (16.58±9.11 *vs*. 18.71±9.11; Z = 2.95; *P* = 0.09; ES: η^2^p = 0.041, 95% CI: 0.00–0.16; Fig 4).

#### Secondary continuous efficacy measures

Data on secondary continuous efficacy measures are presented in Table 4.

**Table 4:** Secondary outcomes time-by-treatment interaction (general linear model) from week 0 to week 20.

Measure	Comparison	Change from baseline	*P* value
**BDI**	**SER *vs*. PLA**	**-7.08 *vs*. -3.48**	**<0.01**
** **	**GCBT *vs*. GPT**	**-6.60 *vs*. -3.74**	**<0.05**
	SER+GCBT *vs*. PLA+GCBT	-8.50 *vs*. -5.03	= 0.05
	**SER+GPT *vs*. PLA+GPT**	**-5.75 *vs*. -1.68**	**<0.01**
HAMD	SER *vs*. LA	-5,23 *vs*. -3.53	= 0.05
	GCBT *vs*. GPT	-4.91 *vs*. -3.76	= 0.19
	SER+GCBT *vs*. PLA+GCBT	-5.88 *vs*. -4.09	= 0.15
	SER+GPT *vs*. PLA+GPT	-4,61 *vs*. -2.89	= 0.16
HAMA	**SER *vs*. PLA**	**-7.18 *vs*. -4.46**	**<0.01**
	GCBT *vs*. GPT	-6.22 *vs*. -5.3	= 0.36
	SER+GCBT *vs*. PLA+GCBT	-7.65 *vs*. -5.02	= 0.09
	**SER-GPT *vs*. PLA+GPT**	**-6.75 *vs*. -3.8**	**<0.05**
CGI-S	**SER *vs*. PLA**	**-1.15 *vs*. -0.61**	**<0.01**
	GCBT *vs*. GPT	-0.96 *vs*. -0.77	= 0.35
	SER+GCBT *vs*. PLA+GCBT	-1.18 *vs*. -0.78	= 0.16
	**SER+GPT *vs*. PLA+GPT**	**-1.14 *vs*. -0.4**	**<0.01**
FNE	SER *vs*. PLA	-3.67 vs. -1.84	= 0.06
	GCBT *vs*. GPT	-3.24 vs. -2.17	= 0.27
	SER+GCBT *vs*. PLA+GCBT	-3.94 vs. -2.66	= 0.34
	SER+GPT *vs*. PLA+GPT	-3.42 vs. -0.89	= 0.08

Last observation carried forward; SER = Sertraline, PLA = Placebo, GCBT = Group Cognitive-Behavioural Therapy, GPT = Group Psychodynamic Therapy. BDI = Beck Depression Inventory; HAMD = Hamilton Depression Rating Scale; HAMA = Hamilton Anxiety Rating Scale; CGI-S = Clinical Global Impression-Severity; FNE = Fear of Negative Evaluation

There was a reduction in FNE scores at week 20 both in patients receiving sertraline (Z = -4.07; *P*<0.001) and placebo (Z = -3.64; *P*<0.001), but general linear model did not reveal a time-by-treatment interaction (FEMM: 21.29±6.98 *vs*. 22.29±6.98; Z = 3.59; *P* = 0.06, ES: η^2^p = 0.024; 95% CI: 0–0.09). Similarly, both GCBT and GPT were associated with significant reduction in FNE scores (Z = -4.57; *P*<0.001 and Z = -3.08; *P*<0.01, respectively), but there was no time-by-treatment interaction (FEMM: 21.37±6.98 *vs*. 22.27±6.99; Z = 1.21; *P* = 0.27, ES: η^2^p = 0.008, 95% CI: 0.00–0.060). The groups SER+GCBT, SER+GPT and PLA+GCBT showed a reduction in FNE scores between week 0 and 20 (all *P*<0.01). There was no difference in time for the PLA+GPT group (*Z* = -1.33; *P* = 0.183). In pairwise comparisons, there was no time-treatment interaction neither for SER+GCBT *vs*. PLA+GCBT (FEMM: 21.12±7.14 *vs*. 21.56±7.15; *Z* = 0.94; *P* = 0.34; ES: η^2^p = 0.013, 95% CI: 0.00–0.10) nor for SER+GPT *vs*. PLA+GPT (FEMM: 21.44±6.86 *vs*. 23.11±6.86; ES: η^2^p = 0.045, 95% CI: 0.00–0.17).

There was a reduction in BDI scores at week 20 both in patients receiving sertraline (*Z* = -6.31; *P*<0.001) and placebo (*Z* = -4.33; P<0.001), with a significant time-by-treatment interaction favouring sertraline (FEMM: 9.26±8.50 *vs*. 10.15±8.50; *Z* = 9.82; *P*<0.01; ES: η^2^p = 0.064, 95% CI: 0.01–0.15). Both GCBT and GPT were associated with significant reduction in BDI scores (*Z* = -6.016 and *Z* = -4.72; *P*<0.001 for both), and there was a significant time-by-treatment interaction favouring GCBT (FEMM: 9.63±8.51 *vs*. 9.82±8.51 *Z* = 6.03; *P* = 0.015; ES: η^2^p = 0.04; 95% CI: 0.001–0.119). There was a trend favouring SER+GCBT over PLA+GCBT (FEMM: 9.15±8.36 *vs*. 10.02±1.36; *Z* = 3.89; *P* = 0.052; ES: η^2^p = 0.05, CI 95%: 0.00–0.08) and SER+GPT was superior to PLA+GPT (FEMM: 9.36±8.77 *vs*. 10.27±8.77; *Z* = 8.56; *P*<0.01; ES: η^2^p = 0.11; 95% CI: 0.01–0.26).

There was a significant reduction in HAMD scores at week 20 both in patients receiving sertraline (*Z* = -5.93; *P*<0.001) and placebo (*Z* = -6.03; *P*<0.001), with a time-by-treatment interaction trend favouring sertraline (FEMM: 6.21±4.69 *vs*. 5,65±4,70; *Z* = 3.84; *P* = 0.052; ES: η^2^p = 0.03, 95% CI: 0.00–0.10). Both GCBT and GPT were associated with significant reduction in HAMD scores (*Z* = -6.21 and *Z* = -5.77; *P*<0.001 for both), but with no time-by-treatment interaction (FEMM: 6.39±4.68 *vs*. 5.42±4,68; *Z* = 1.74; *P* = 0.19; ES: η^2^p = 0.01, 95% CI 95%: 0.00–0.07). Neither SER+GCBT was superior to PLA+GCBT (FEMM: 6.35±4.65 *vs*. 6.42±4.65; *Z* = 2.12; *P* = 0.15; ES: η^2^p = 0.028, 95% CI: 0.00–0.14) nor SER+GPT was superior to PLA+GPT (FEMM: 6.08±4.73 *vs*.4.74±4.73; *Z* = 1.494; *P* = 0.22; ES: η^2^p = 0.03, 95% CI: 0.00–0.14).

There was a significant reduction in HAMA scores at week 20 both in patients receiving sertraline (*Z* = -6.20; *P*<0.001) and placebo (*Z* = -5.92; *P*<0.001), with a significant time-by-treatment interaction favouring sertraline (FEMM: 7.53±5.41 *vs*. 8.53±5.41; *Z* = 7.669; *P* = 0.006; ES: η^2^p = 0.05, 95% CI: 0.00–0.13). Both GCBT and GPT were associated with significant reduction in HAMA scores (*Z* = -6.02 and *Z* = -6.28, respectively; *P*<0.001 for both), without time-by-treatment interaction (FEMM: 8.41±5.42 *vs*. 7.66±5.42; *Z* = 0.83; *P* = 0.36; ES: η^2^p = 0.01, 95% CI 95%: 0.00–0.05). There was also no difference between SER+GCBT and PLA+GCBT (FEMM: 7.53±5.74 *vs*. 9.15±5.58; *Z* = 2.93; *P* = 0.09; ES: η^2^p = 0.04, 95% CI: 0.00–0.153). SER+GPT was superior to PLA+GPT (FEMM: 7.53±5.26 *vs*. 7.80±5.27; *Z* = 5.75; *P*<0.05; ES: η^2^p = 0.08, 95% CI: 0.00–0.21).

There was a significant reduction in CGI-S scores at week 20 both in patients receiving sertraline (*Z* = -5.67; *P*<0.001) and placebo (*Z* = -4.44; *P*<0.001), with a significant time-by-treatment interaction favouring sertraline (FEMM: 3.39±1.22 *vs*. 3.92±1.23; *Z* = 8.18; *P*<0.01; ES: η^2^p = 0.054, 95% CI: 0.01–0.14). Both GCBT and GPT were associated with significant reduction in CGI-S scores (*Z* = -5.48 and *Z* = -4.76 respectively; *P*<0.001 for both), but there was not time-by-treatment interaction (FEMM: 3.64±1.26 *vs*. 3.69±1.26; *Z* = 0.88; *P* = 0.35; ES: η^2^p = 0.01, 95% CI: 0.00–0.05). There was not a time-by-treatment interaction for SER+GCBT and PLA+GCBT (FEMM: 3.35±1.28 *vs*. 3.88±1.28; *Z* = 2.05; *P* = 0.16; ES: η^2^p = 0.03, 95% CI: 0.00–0.13). SER+GPT was superior to PLA+GPT (FEMM: 3.42±1.18 *vs*. 3.97±1.18; *Z* = 7.54; *P*<0.01; ES: η^2^p = 0.10, 95% CI: 0.03–0.24).

### Adverse events

No clinically significant adverse events were observed more frequently in the sertraline group than in the placebo group or in GCBT than in the GPT group.

## Discussion

Partially supporting our main hypothesis, the present study data confirm that combining sertraline and psychotherapy is superior to psychotherapy alone, however not in all measures. In addition, the differences between the treatments were subtle, and even when they were significant, the effect size was small. Effect sizes may be underestimated because there was no comparison group without any kind of active treatment, such as a waiting list or pill placebo group.

Although there was improvement in all groups, sertraline plus GCBT or GPT was superior to GCBT or GPT plus pill placebo in many outcomes. Patients allocated to receive sertraline had a greater probability of achieving remission, greater probability of significant response of social anxiety symptoms, greater reduction in SADS, BDI, HAMA and CGI-S scores. These findings suggest an additive or synergistic effect of these treatment modalities. In divergence with these findings, sertraline plus psychotherapy (GCBT or GPT) was neither superior in the acquisition of social skills (as measure by the M-MMSE) nor in the reduction of FNE score in comparison to psychotherapy alone.

Our study adds to a literature with mixed results, suggesting possible benefits of combined treatment over pharmacotherapy or psychotherapy alone for anxiety disorders in general and SAD in particular. Our results contrast with the study by Davidson et al. [29] who had conducted a trial (N = 295) to examine improvement in response rates with the addition of fluoxetine to CBT and had found that the combined treatment did not yield any further advantage over CBT alone. Similarly, Blomhoff et al. [30] compared the efficacy of brief exposure treatment conducted by primary care physicians with sertraline plus exposure therapy in generalized social phobia. A total of 387 patients were allocated to receive sertraline or placebo and separately to exposure or general medical care. Sertraline was associated with greater efficacy than placebo, whereas exposure alone was not. They observed only a trend (*P* = 0.059) in favour of combined treatment (sertraline plus exposure therapy). The same group examined the efficacy of these treatments one year after their initiation. The four treatment groups had showed improvements in efficacy measures from baseline to week 52. On the other hand, patients who had been treated with exposure therapy and placebo had further improvements in social anxiety symptoms during the follow-up period, whereas patients who had received sertraline–either alone or in combination with exposure therapy–had no further improvement after the end of the treatment period, possibly for catching up the lack of initial efficacy [31].

On the other hand, the present study results are in accordance with Blanco et al. [32] who compared phenelzine, CBGT and their combination for SAD and found combined phenelzine and CBGT treatment to be superior to either treatment alone and to placebo. Knijnik et al. [11] studied 58 SAD patients submitted to 12 weeks of psychodynamic group therapy (PGT) plus clonazepam or clonazepam alone. The authors showed that patients who had received PGT plus clonazepam presented significantly greater improvement than those who had received only clonazepam. These results may not be comparable to ours since benzodiazepines do not act primarily at serotonergic system. Walkup et al. [33], in a sample of 488 children between the ages of 7 and 17 years old who had a primary diagnosis of separation anxiety disorder, generalized anxiety disorder, or social phobia, found that a combination of CBT and sertraline had a superior response rate than CBT or sertraline alone. Barlow et al. [34], in a treatment trial for panic disorder comparing CBT, imipramine and their combination, found combined treatment superior to either in monotherapy.

Finally, our findings of the superiority of combined treatment are also according to meta-analyses that have shown the superiority of combined treatment over monotherapies in diverse anxiety disorders [17], [18], [19].

Taken together, the available evidence seems to support the superiority of combined treatment over psychotherapy alone for the treatment of SAD.

As a limitation, due to the lack of a sertraline plus wait list group or other inactive treatment, we could not evaluate if combining sertraline and psychotherapy would be superior to sertraline alone.

Comparing GCBT and GPT, we observed few significant differences in favour of GCBT. GCBT was superior in the acquisition of social skills (M-MMSE) and in the reduction of self-reported depressive symptoms (BDI). However, no other differences were observed. This lack of other differences between GCBT and GPT is not due to the lack of effectiveness since there were improvement in both groups. This is in concordance with Barkowski et al. [35] who had demonstrated that group psychotherapy for SAD is an efficacious treatment.

The lack of clear superiority for GCBT in this trial might be due to some different factors. GPT were conducted by experienced therapists who were enthusiasts of their techniques and could not be considered ‘placebo therapy’. As shown by Munder et al. [36], investigator allegiance heavily influences results of psychotherapy outcome studies. Our trial controlled for an investigator allegiance effect by including experts from the two approaches. In GCBT, social skills training was added to exposure therapy and cognitive restructuring while in the GPT those techniques were formally absent. GPT may have provided opportunities of exposing and modelling due to the group format. It is also possible that the relatively equity of GCBT and GPT could be due to nonspecific psychotherapy effects such as expectancies of improvement. Different from CBT, that has a large body of research showing benefits for patients with social anxiety disorder [5], [37], psychodynamic therapy is much less researched but it is frequently used [38].

Our results are somewhat similar to the only head-to-head comparison of CBT and psychodynamic therapy to our knowledge. Leichsenring et al. [10] randomly assigned 495 outpatients with SAD to manual-guided CBT (N = 209), manual-guided psychodynamic therapy (N = 207), or a waiting list condition (N = 79). Remission rates were significantly superior in the CBT (36%) and psychodynamic therapy (26%) groups than in waiting list group (9%) as well as response rates (CBT = 60%, psychodynamic therapy = 52%, waiting list = 15%). CBT and psychodynamic therapy were superior to waiting list for both remission and response. CBT was significantly superior to psychodynamic therapy for remission but not for response.

Altogether, these studies data and the present study data are consistent with previous meta-analysis [37], [5] that concluded that CBT is the therapy of choice for SAD but psychodynamic therapy is a viable option depending on the patient/therapist preference.

To our knowledge, this is the first study to examine whether GCBT or GPT have different additive or synergistic effects with a SSRI on SAD.

Sertraline did not change the probability of remission neither when combined with GCBT nor with GPT. There were also no significant differences in the probability of response of social anxiety symptoms when combined with GCBT (17.6% *vs*. 7.7%, *P* = 0.197), but SER-GPT had a superior probability of response of social anxiety symptoms in comparison to PLA-GPT (33.3%, *vs*. 11.4%, *P*<0.05). The synergistic effect of sertraline and GCBT appears clearer in the primary measures of social skills acquisition and in SASD. SER+GCBT had a superior improvement in M-MSSE in comparison with PLA+GCBT (*P*<0.01) and there was no difference between SER+GPT vs PLA+GPT (*P* = 0.80; Fig 3). Interestingly, there were no differences in final M-MSSE scores between sertraline and placebo when GCBT and GPT are compared jointly (*P* = 0.437). A greater reduction of SADS score was observed for SER+GCBT in comparison with PLA+GCBT (*P*<0.05; Fig 3), but not for SER+GPT versus PLA+GPT (*P* = 0.09; Fig 3). Diversely, sertraline appears to have a more synergistic effect when combined with GPT on secondary measures. In self-reported depressive symptoms (BDI), there was only a trend favouring SER+GCBT over PLA+GCBT (*P* = 0.052) while SER+GPT was clearly superior to PLA+GPT (*P*<0.01). The same was observed related to anxiety symptoms (HAMA). In this measure, there was no difference between SER+GCBT and PLA+GCBT (*P* = 0.091) but SER+GPT was superior to PLA-GCBT (*P*<0.05). Sertraline had also affected more the severity of symptoms when combined with GPT. In CGI-S, there was no difference for SER+GCBT and PLA+GCBT (*P* = 0.157) while SER+GPT was superior to PLA-GPT (*P*<0.01).

As discussed above, GCBT by itself was superior to GPT in the acquisition of social skills and in the reduction of self-reported depressive symptoms. Thus, we hypothesized that the incremental acquisition of social skills represents a truly synergistic effect while the difference in depressive symptoms could be related to the reduced efficacy of GPT in this measure while sertraline has a recognized efficacy in depression. According to Graeff’s model [15, 39], 5-HT would enhance learned responses to distal threat while inhibiting unconditioned responses (e.g., primitive fixed action patterns) to proximal threat by inhibiting the periaqueductal gray matter activity. Tolerance to aversive states, as required in exposure therapy, may depend on an appropriate 5-HT action and a low 5-HT availability may be associated with a lower ability to learn alternative skills to cope with these situations (14). Some conceptualizations of SAD consider that patients possess adequate social skills but their ability to focus on social interactions and use the skills appropriately are hindered by the aversive nature of anxiety [40, 41].

The present study had limitations. Psychological treatments were provided by experts and may show lower efficacy in less specialized settings. Individuals who dropped out from the study post randomization but prior to receiving any treatment could not be included in the analyses. Additionally, this sample was composed of treatment-seeking patients with some restrictions at the enrolment phase (e.g., no severe depressive symptoms). Therefore, our results may not be fully generalized to all patients with SAD. As in many clinical trials, response was defined as a standardized reduction in a predefined symptom severity rating scale. Therefore, patients considered responders may still have distressing residual symptoms. Because self-exposure was neither assessed nor discouraged in GPT, it is possible that spontaneous exposure occurred, which may have contributed to patient's improvement. Our study had a relatively small sample size, limiting our power to detect significant results in certain measures or to use more complex statistical models including controlling for multiple comparisons. Our study lasted for only 20 weeks, a relatively short treatment period for a condition as chronic as SAD. It is possible that longer periods of psychotherapy would be needed to obtain the full benefit of GCBT or GPT. Despite these limitations, our study suggests that the addition of sertraline to psychotherapy may be beneficial on many SAD aspects. Future studies with larger samples may be necessary before definitive prescriptive recommendations are made.

## Conclusions

Our study provides some empirical support for the use of combined treatment for SAD since sertraline plus psychotherapy were superior to psychotherapy alone in diverse measures. We also observed that GCBT might be superior to GPT in some secondary measures what is compatible with the view that GCBT is the therapy of choice for SAD. On the other hand, group psychodynamic therapy is a viable option depending on patient or therapist preference. Additionally, sertraline potentiated the efficacy of GCBT by enhancing social skills acquisition and specific social phobia symptoms while the potentiation of GPT by sertraline is clearer in depressive and unspecific anxiety symptoms.

## Supporting information

S1 File Complete dataset. (XLSX)

S2 File CONSORT Checklist Sertraline and Psychotherapy in SAD. (DOC)

S3 File Protocol—English version. (DOCX)

S4 File Protocol—Portuguese version. (DOCX)
