# Supplementary material for: Concomitant treatment with sertraline and social skills training improves social skills acquisition in social anxiety disorder: A double-blind, randomized controlled trial
Source: PLoS One. 2018 Oct 29;13(10):e0205809. doi: 10.1371/journal.pone.0205809 (PMC6205595; doi:10.1371/journal.pone.0205809)
Supplement: S3 File — (DOCX) [file pone.0205809.s003.docx]

###### RESEARCH PROJECT

A multidisciplinary trial to be carried out by Ambulatório de Ansiedade do Instituto de Psiquiatria (AMBAN-IPQ) do Hospital das Clínicas da Faculdade de Medicina da Universidade de São Paulo (HC-FMUSP) to evaluate the efficacy of selective serotonin reuptake inhibitors (SSRI) alone or in combination with cognitive behavioural therapy (CBT) in the short-term and continuation treatment of Social Phobia.

**1 Introduction**

1.1 Background and Diagnosis

Social phobia was described for the first time by Marks and Gelder (1966) and included as a discrete nosological entity in DSM-III in 1980. Since then, an increasing body of knowledge on the subject has been developed. DSM-III (APA, 1980) defined social phobia as fear of or avoidance of any kind of public performance, such as speaking, eating, drinking or writing in front of other people, or using a public lavatory, and so on. Avoidance personality disorder, a condition with similar profile, was defined as hypersensitivity to rejection, problems in establishing social relations, avoidance of social situations and diminished self-esteem. The generalized subtype of social phobia was introduced with the publication of DSM-III-R (APA, 1987), with considerable overlap with avoidance personality disorder. IDC-10 (WHO, 1989) followed the same standards of DSM-III-R, but included avoidance of social situations as a necessary condition for establishing the diagnosis. The most recent diagnosis criteria set introduced some changes: according to DSM-IV (APA, 1994) the essential feature of avoidance personality disorder is continuous and marked fear of social situations, in which patients may be in contact with strangers or may be scrutinized by others; thus, the fear of humiliation and symptoms and signs of anxiety that lead to embarrassment or constrain are common. Patients realizes that the fear is excessive or irrational. Social situations and of performance are avoided or undergone with much anxiety and discomfort.

1. Comorbidity

Comorbidity with depression is frequent. There is a study showing an incidence of 70% of Major Depression among social phobics. (Van American et al., 1991). In another study, Cox et al. (1994) reported suicidal attempts by 12% of patients with a diagnosis of social phobia, although Major Depression was not found. Abuse of alcohol and other psychoactive substances were also frequent (Lotufo-Neto; Gentil, 1994 and Sussaman, 1993). Other disorders that may be associated to social phobia are anxiety and eating disorders and personality disorders, such as dependent, esquizotypic and paranoid personality disorders (Alnaes, Torgersen, 1988)

1. Response to specific pharmacological treatment

Some studies have evaluated the response to treatment with MAO inhibitors (Versiani et al., 1992 and Fahlen, 1995) in which phenelzine as well as moclobemide and brofaromine proved to be effective in reducing social anxiety. Other studies, such as Davidson et al.’s (1993) showed a good response to benzodiazepines. There are few studies, however, with SSRIs. Most of these studies are case reports and open studies (Van American et al., 1994; Lepola et al., 1994 and Mancini; Van Ameringen, 1996). The table below summarizes the results of these studies:

| AUTHORS | YEAR | N | DRUG | OUTCOME |
| --- | --- | --- | --- | --- |
| Van Ameringen et al | 1994 | 22 | Sertralina | 16 (80%) responders |
| Lepola et al. | 1994 | 3 | Citalopram | All responders |
| Mancini; Van Ameringen | 1996 | 18 | Paroxetina | 15 (83,3%) responders |

The few controlled studies with SSRIs had small sample size (Van Vliet et al., 1994 e Katzelnick et al., 1995). Van Vliet et al had compared fluvoxamine and placebo in 33 patients with social phobia showing that those who received fluvoxamine had a significant better outcome than those who received placebo (46% x 7%, respectively). However, there was no significant difference regarding phobic avoidance. Katzelnick et al evaluated 12 patients with social phobia. Six of them received sertraline in flexible doses (up to 200mg daily) and the other six had received placebo. The groups differed significantly with 50% of those who received sertraline showing better outcome in Liebowitz anxiety scale versus 9% in placebo group. The small sample size makes generalization difficult.

Therefore, the efficacy of SSRI remains uncertain both in acute treatment and in maintenance treatment in patients with social phobia.

1. Response to behavioural and cognitive therapy

Many behavioural and cognitive techniques were proposed and reported as effective for treating social phobia, among which, social skills training, exposure therapy, cognitive based therapy and cognitive behavioural group therapy. Among the proposed techniques, in vivo exposure to feared situations is the technique mostly recognized as central and effective for reducing phobic anxiety reactions (Barlow, 1988). Many projects have studied exposure therapy efficacy in the treatment of social phobia (Al-Kubaisy et al., 1992; Alstrom et al., 1984, Turner et al., 1994 and Wlaslo et al., 1990).

Results are usually quite incomplete, nevertheless, when compared to results obtained with agoraphobic patients, for example.

There are also not many studies comparing the effectiveness of behavioural techniques to pharmacological treatments or treatments based on psychodynamic psychotherapy.

A consensus is now being developed about the usefulness of drug treatment in social phobia, but there is no study on the possible additional benefits of cognitive-behavioural therapy on patients treated with SSRIs.

**2 Objectives**

1. Evaluate the efficacy and safety of a selective serotonin reuptake inhibitor (SSRI) in comparison to placebo in the 8-week short-term treatment of patients with a primary diagnosis of social phobia.
2. Evaluate if adding behavioral therapy (CBT) to pharmacological treatment (conventional clinical management) brings any additional benefits in the treatment of these patients during a 20-week follow-up.
3. Evaluate the efficacy of CBT in comparison to psychodynamic therapy in patients with primary diagnosis of social phobia.
4. Evaluate the effect of treatments on social skills acquisition in each of the groups.

**3 Methods**

1. Subjects
2. Inclusion criteria

- 128 patients will be included from the Anxiety Outpatient Clinic of the Psychiatry Institute of HC-FMUSP
- age between 18 and 65 years
- diagnosis of Social Phobia (DSM-IV), of at least one year, by a psychiatrist using the Structured Clinical Interview for DSM-IV (First et al, 1996)
- scores equal or above 4 on the Global Clinical Impression Scale (CGHI, Guy, 1976) and equal or above 17 and 24 in SAD and FNE (Watson; Friend, 1969), respectively.

1. Exclusion Criteria

- Major depression diagnosis with suicidal risk or BDI (Beck, 1961) equal or above 30 or HAMD score (Hamilton, 1960) equal or above 21
- any other primary psychiatric diagnosis from DSM-IV, except Social Phobia and Avoidant Personality Disorder
- organic disorder, epilepsy
- Alcohol intake above 2 units/day
- patients on anti-depressant drugs and benzodiazepines

1. Outcome measures
2. Evaluation of anxiety symptoms:

- Hamilton Anxiety Scale (HAMA; HAMILTON, 1960)

1. Evaluation of depressive symptoms

- Hamilton Depression Scale (HAMD; HAMILTON, 1959)
- Beck Depression Inventory (BDI; BECK et al., 1961)

1. Evaluation of severity of social-phobic symptoms

- Social Avoidance and Distress scale (SAD; WATSON; FRIEND, 1969)
- Fear of Negative Evaluation scale (FNE; WATSON; FRIEND, 1969)

1. Baseline and repeated global evaluation

- Clinical Global Impression (CGI; GUY, 1976)

3.2.5 Evaluation of social skills

- Multidimensional Scale of Social Expression – Motor Part (M-MSSE, Caballo, 1993)

1. Experimental Planning

The study will be performed with 4 groups with 32 patients in each group. Patients in group I, will receive active drug and group behavioural therapy, so that it is possible to evaluate the addition of CBT to SSRI; in group II, patients will receive active drug and group psychodynamic therapy, and the effect of SSRI independently of CBT can be evaluated; in group III, placebo and Group Behavioural Therapy will be administered, and the effect of CBT can be evaluated, and group IV will receive psychodynamic therapy and placebo, and the nonspecific effects of the treatments can be evaluated.

Each therapeutic group will be composed of 8 patients, and each cell of the experimental design will have 4 groups of psychotherapy. The 128 patients will be randomly assigned to one these groups. Concomitantly, patients will be randomly divided to receive sertraline or placebo. The experimental design can be viewed in Table 1.

| Table 1. Experimental design | |
| --- | --- |
| Group I – 32 subjects  Cognitive behavioural therapy  Sertraline | Group III – 32 subjects  Cognitive behavioural therapy  Placebo |
| Group II – 32 subjects  Psychodynamic therapy  Sertralina | Group IV – 32 Subjects  Psychodynamic therapy  Placebo |

3.4 Procedures and evaluation

Patients will be screened at the anxiety clinic (Ambulatório de Ansiedade; AMBAN) and referred for a diagnostic interview and physical examination with a psychiatrist. During this interview (week -2), patients will be evaluated for diagnostic confirmation, will complete a consent form and will received 2 weeks of daily placebo capsule (lactose) for wash-out.

Next, they will be sent to the psychological evaluation, which includes the instruments of evaluation of personality, social skills, quality of life. Later, the patients will be allocated to the therapeutic groups. Patients who meet diagnostic criteria will be randomized to one of 4 treatment groups. The treatment will last for 20 weeks.

Next, patients will be sent to psychological evaluation, which includes the instruments of evaluation of social skills. After that, patients which fulfil inclusion criteria will be randomized to one of 4 combined treatment groups. The treatment will last for 20 weeks.

Patients who score below 17 in SAD and less than 24 in FNE at the end of the wash-out period, will be referred to the AMBAM didactic outpatient clinic for open treatment and excluded of the study.

At any point in the study, if BDI score exceeds 30 points and HAMD score is greater than 21 points, it will be discussed with the team to evaluate each case and how to proceed.

Sertraline and Placebo will be given in identical capsules. Wash-out phase placebo will be provided in a separate bottle labelled as Bottle 0. In the double-blind phase, bottles will be numbered from Bottle 1 to Bottle 20.

The evaluation and follow-up sessions will be performed at weeks 0, 1, 2, 4, 8, 12, 16 and 20 for the 4 treatment groups, lasting 45 minutes each.

Once the optimal dose (most effective and tolerable dose) is achieved, it should be maintained unless side effects which require a reduction or withdrawal of the medication occur. The physician should opt for an initial reduction to the previous dose before discontinuing medication.

Doses should be standardized according to the figure below:

|  |  |  |  |  |  |  |  |
| --- | --- | --- | --- | --- | --- | --- | --- |
|  |  |  |  |  | **+3** | **+3** | **+3** |
|  |  |  |  | **+2** | **+2** | **+2** | **+2** |
|  |  |  | **+1** | **+1** | **+1** | **+1** | **+1** |
|  |  | **PD** | **PD** | **PD** | **PD** | **PD** | **PD** |
| **Week -2** | **PRÉ - SD**  **Week 0** | **Week1** | **Week 4** | **Week 8** | **Week 12** | **Week 16** | **Week 20** |
| **Wash out** | **visit 0** | **visit 1** | **Visit 2** | **visit 3** | **Visit 4** | **visit 5** | **Visit 6** |

SD- defined standard dose (50 mg of sertraline). Patients will receive a dose varying from 1 to 4 times the standard dose.

+1 - 1^st^ dose increase option

+2 - 2^nd^ dose increase option

+3 - 3^rd^ dose increase option

Patients in groups I and III will receive 20 sessions of 90 minutes once a week of group cognitive behavioural therapy (CBT). A Researcher's Manual was developed assuring that therapists have a standardized procedure. Patients in groups II and IV will receive 20 sessions of 90 minutes of group psychodynamic therapy. Psychodynamic therapy was defined as therapeutic support in which the professional will not give exposure instructions and will not perform social skills training. It will be an occasion when the patient can exchange experiences through testimonials and in a manner that nonspecific factors of the therapy will be present. The course of action of these therapists was pre-determined, and a manual of action was elaborated.

Therapy sessions will be recorded. The analysis of the content of the sessions will be done by 2 evaluators who will listen to 30% of the tapes, chosen randomly.

3.5 Statistical analysis

The null hypotheses are:

- There is no clinical difference between SSRI and placebo for short-term treatment of social phobics

-Adding CBT does not improve treatment with SSRI in 20 weeks in these patients

- There is no difference in social skills acquisition between groups

Subject's last observation carried forward data will be used for data analysis. All subjects with baseline measures and at least one follow-up measurement will be included in the intent-to-treat sample.

Kolmogorov-Smirnov test and Levene’s test will be used to evaluate normality of distribution and homogeneity of variances, respectively, prior to any statistical testing. Independent samples t-tests will be used to examine differences between groups. Mann-Whitney Test will be used to examine variables that not follow a normal distribution. Categorical data will be compared using Chi-square test and Fisher’s exact test. A multifactorial ANOVA with repeated measures will used to examine changes over time between treatment groups. Pairwise comparisons planned are: sertraline vs. placebo; cognitive behavioural therapy vs. psychodynamic therapy; Group I vs. Group III; Group II vs. Group IV. Alpha value was set at 0.05 for significance. When needed, alpha was adjusted for multiple comparisons.

3.6 Ethics

The project complies with Resolution # 196 of October 10, 1996 of the National Health Council (CONSELHO NACIONAL DE SAÚDE).

All patients will be informed of the objectives and methodology of the experiment and will fill informed consent forms.

**References:**

AL-KUBAISY, T.; MARKS, I.M.; LOOSDAIL, S. Role of exposure homework

in phobia reduction: a controlled study. Behavior Therapy, v.23, p.599-621, 1992.

ALNAES, R.; TORGERSEN, S. The relationship between DSM-III

symptom disorders (Axis I) and personality disorders (axis II) in an

outpatient population. **Acta Psychiatr. Scand.,** v.78, p.485-92, 1988.

ALSTROM, J.E.; NORDLUND, C.L.; PERSSON, G. The effects of four

treatment methods on social phobic patients not suitable for insigth-oriented psychoterapy. **Acta Psychiatrica Scandinavica,** v.70, p.97-110, 1984.

AMERICAN PSYCHIATRIC ASSOCIATION. **Diagnostic and**

**statistical manual of mental disorders**, 3. ed. Washington, DC, American Psychiatric Association, 1980.

AMERICAN PSYCHIATRIC ASSOCIATION. **Diagnostic and**

**statistical manual of mental disorders**, 3. ed. rev. Washington, DC, American Psychiatric Association, 1987.

AMERICAN PSYCHIATRIC ASSOCIATION. **Diagnostic and**

**statistical manual of mental disorders,** 4. ed. Washington, DC, American Psychiatric Association, 1994.

BARLOW, D.H. **Anxiety and its disorders**. New York, Guilford, 1988.

CABALLO VE. Manual de Evaluación y Entrenamiento de las Habilidades Sociales. Spain: Siglo XXI de España; 1993

COX, B.J.; DIRENFELD, D.M.; SWINSON, R.P.; NORTON, G.R.

Suicidal ideation and suicidal attempts in panic disorders and social phobia. **Am. J. Psychiatry,** v.151, p.882-7, 1994.

DAVIDSON, J.R., POTTS, N., RICHICHI, E., KRISHNAN, R., FORD, S.M.,

SMITH, R., WILSON, H.T. Treatment of social phobia with clonazepan and placebo. **J. Clin. Psychopharmacology,** v.13, p.423-8, 1993.

FIRST MB, SPITZER, ROBERT L, GIBBON MIRIAM, AND WILLIAMS, JANET B.W. Structured Clinical Interview for DSM-IV Axis I Disorders, Clinician Version (SCID-CV). . Washington, D.C.: **American Psychiatric Press**, Inc; 1996.

GUY, W ECDEU assessment manual for psychopharmacology, revised NIMH,

Publishers, Bethesda

FAHLEN, T. Personality traits in social phobia. II: Changes during

treatment. **J. Clin. Psychiatry,** v.56, p.569-73, 1995.

HAMILTON, M. The assessment of anxiety states by rating. **British Journal of**

**Medical Psychology**, v.32, p.50-55, 1959.

HAMILTON, M A rating scale for depression. **J. Neurol Neurosurg**

**Psychiatry**, v.23, p. 56-62, 1960.

KATZELNICK, D.J.; KOBAK, K.A.; GREIST, J.H.; JEFFERSON, J.W.;

MANTLE, J.M.; SERLIN, R.C. Sertraline in social phobia: a double-blind placebo-controlled crossover study. **Am. J. Psychiatry,** v.152, p.1368-71, 1995.

LEPOLA, U.; KOPONEN, H.; LELNONEN, E. Citalopran in the treatment of

social phobia: a report of three cases. **Pharmacopsychiatry.** v.27, p.186-8, 1994.

LOTUFO-NETO, F.; GENTIL, V. Alcoholism and phobic anxiety- a

clinical-demographic comparison. **Addiction,** v.89, p.447-53, 1994.

MANCINI, C.; VAN AMERINGEN, M. V. Paroxetine in social phobia. **J.Clin. Psychiatry.** v.57, p.519-22, 1996.

SUSSMAN, N. Treating anxiety while minimizing abuse and

dependence. **J. Clin. Psychiatry,** v.54, p.44-51, 1993. Supplement 5.

TURNER, S.M.; BEIDEL, D.C.; JACOB, R. Social phobia: a comparison of

behavior therapy and atenolol. **J.Consult. Clin. Psychol.,** v.62, p.350-358, 1994.

VAN AMERINGEN, M.; MANCINI, C.; STYAN, G.; DONISON, D.

Relationship of social phobia with other psychiatrc illness. **J. Affect. Disord.,** v.21, p.93-9, 1991.

VAN AMERINGEN, M.; MANCINI, C.; STREINER, D. L.; Sertraline in social

phobia. **J. Affect Dis.,** v.31, p.141-5, 1994.

VAN VLIET, I.M.; DEN BOEF, J.A.; WESTENBERG, H.G.;

Psychopharmacological treatment of social phobia; a double blind placebo controled study with fluvoxamine. **Psychopharmacology,** v.115, p.128-34, 1994

VERSIANI, M.; NARDI, A.E.; MUNDIM, F.D.; ALVES, A.D.;

LIEBOWITZ, M.R.; AMREIN, R. Pharmacoterapy of social phobia. A controlled study with moclobemide and phenelzine. **Br. J. Psychiatry,** v.161, p.353-60, 1992.

WATSON, D.; FRIEND, R. Measurement of social-evaluation anxiety. **J.**

**Consult**. **Clin. Psychol**., v. 33, p. 448-457, 1969

WLASLO, Z.; SCHOEREDER-HARTWIG, K.; HAND, I. Exposure in vivo

versus social skills training for social phobia: Long-term outcome and differential effects. **Behav. Res. Therapy,** v.28, p.181-193, 1990.

WORLD HEALTH ORGANIZATION. **Mental disorders: glossary**

**and guide to their classification in accordance with the 9th revision of I.C.D.**  Geneva, World Health Organization, 1978.

WORLD HEALTH ORGANIZATION. **International classifications of**

**diseases.** 10. rev. Geneva, World Health Organization, 1989
