# Supplementary material for: Concomitant treatment with sertraline and social skills training improves social skills acquisition in social anxiety disorder: A double-blind, randomized controlled trial
Source: PLoS One. 2018 Oct 29;13(10):e0205809. doi: 10.1371/journal.pone.0205809 (PMC6205595; doi:10.1371/journal.pone.0205809)
Supplement: S4 File — (DOCX) [file pone.0205809.s004.docx]

**Protocolo de pesquisa**

Projeto interdisciplinar a ser realizado pelo Ambulatório de Ansiedade do IPQ do HC-FMUSP sobre avaliação da eficácia da sertralina, da TCC em grupo e do tratamento combinado em pacientes com diagnóstico de Fobia Social.

Autores:

**1 Introdução**

1.1 Histórico e diagnóstico

A fobia social foi descrita pela primeira vez por Marks e Gelder (1966) e incluída no DSM-III em 1980. Desde então, um número crescente de trabalhos sobre o tema vem aparecendo na literatura. O DSM-III (APA, 1980), caracterizou a fobia social como um medo ou esquiva de situações de desempenho como falar em público, comer, beber e escrever diante das pessoas e usar banheiro público, etc., enquanto que a hipersensibilidade à rejeição, dificuldade de relacionamento social, esquiva de situações sociais e auto estima diminuída caracterizavam o transtorno evitador de personalidade. Com a publicação do DSM-III-R (APA, 1987), foi criado o subtipo generalizado de fobia social, havendo considerável sobreposição com o transtorno evitador de personalidade. A CID-10 (WHO, 1989) segue um padrão semelhante ao do DSM-III-R, mas inclui a esquiva de situações sociais como condição necessária para se fazer o diagnóstico. De acordo com o DSM-IV (APA, 1994), a característica essencial do transtorno é um medo acentuado e persistente de situações sociais ou de desempenho na qual a pessoa é exposta a pessoas estranhas ou à possível avaliação por parte delas, temendo a humilhação ou mostrar sintomas de ansiedade, o que levaria à vergonha ou constrangimento. A pessoa reconhece que o medo é excessivo ou irracional. As situações sociais e de desempenho são evitadas ou suportadas com muita ansiedade e desconforto.

1.2 Comorbidade

Comorbidade com depressão é frequente e há um estudo que evidencia prevalência de 70% de depressão maior entre fóbicos sociais, (Van American et al., 1991). Em outro estudo, Cox et al. (1994), embora não se observasse o diagnóstico de depressão maior, tentativas de suicídio foram relatadas por 12% dos pacientes com diagnóstico de fobia social. Também são frequentes o abuso e dependência de álcool e outras substâncias psicoativas (Lotufo-Neto; Gentil, 1994 e Sussaman, 1993). Outros transtornos que podem ser encontrados em comorbidade são: transtornos ansiosos e alimentares e transtornos de personalidade, como o transtorno dependente, esquizotípico e paranoide (Alnaes; Torgersen, 1988).

1.3 Resposta a tratamentos farmacológicos específicos

Há estudos que avaliaram a resposta à terapêutica com o uso de IMAOs (Versiani et al., 1992 e Fahlen, 1995), nos quais tanto a fenelzina, como a moclobemida e a brofaromina mostraram-se eficazes na diminuição da ansiedade social. Outros estudos, como o de Davidson et al. (1993) evidenciaram boa resposta com o uso de benzodiazepínicos. Há poucos estudos, entretanto, com o uso de ISRS. A maioria deles refere-se a relatos de casos e estudos abertos (Van Ameringen et al., 1994; Lepola et al., 1994 e Mancini; Van Ameringen, 1996). A tabela abaixo resume os resultados desses estudos:

| AUTORES | ANO | N | DROGA | RESULTADO |
| --- | --- | --- | --- | --- |
| Van Ameringen et al | 1994 | 22 | Sertralina | 16 (80%) responderam |
| Lepola et al. | 1994 | 3 | Citalopram | Boa resposta nos 3 casos |
| Mancini; Van Ameringen | 1996 | 18 | Paroxetina | 15 (83,3%) responderam |

Os poucos estudos controlados feitos com ISRS utilizaram amostras pequenas (Van Vliet et al., 1994 e Katzelnick et al., 1995). O estudo de Van Vliet comparou a fluvoxamina a um placebo em 33 pacientes com fobia social mostrando que o grupo de pacientes que recebeu fluvoxamina teve melhora significativamente mais acentuada em relação ao grupo placebo (46% x 7%, respectivamente). No entanto, ao se avaliar a esquiva fóbica, a diferença não atingiu nível significativo. O estudo de Katzelnick utilizou 12 pacientes com fobia social. Seis receberam sertralina em doses flexíveis (até 200mg/dia) e os outros 6 receberam placebo. Houve diferença significativa entre os dois grupos, sendo que 50% dos pacientes que receberam sertralina apresentaram melhora na LSAS (Escala de ansiedade social de Liebowitz) contra 9% do grupo que recebeu placebo. A amostra utilizada, no entanto, é muito reduzida, sendo difícil de se generalizar os resultados.

Portanto, permanece aberta, ainda, a questão da eficácia dos ISRS no tratamento agudo e de manutenção em pacientes com fobia social.

- 1. Resposta à terapia comportamental e cognitiva

Diversas técnicas comportamentais e cognitivas foram propostas e divulgadas como eficazes para o tratamento da fobia social, entre elas: o treinamento de habilidades sociais; terapia baseada em exposição; terapia de base cognitiva e terapia comportamental cognitiva em grupo. Das técnicas propostas, a exposição em vivo às situações temidas é a técnica mais largamente reconhecida como central e eficaz na redução das reações de ansiedade fóbica, Barlow (1988). Diversos estudos examinaram a eficácia da exposição, em sua forma pura, no tratamento da fobia social (Al-Kubaisy et al., 1992; Alstrom et al., 1984; Turner et al., 1994 e Wlaslo et al., 1990).

Os resultados, porém, costumam ser bastante incompletos, quando comparados, por exemplo, com os resultados obtidos com pacientes agorafóbicos.

Faltam também estudos que comparem a eficácia das técnicas comportamentais com aquelas de tratamentos farmacológicos ou tratamentos com base em psicoterapia psicodinâmica.

Vem se desenvolvendo um consenso sobre a utilidade do tratamento medicamentoso na fobia social, mas não existem estudos sobre benefícios da associação de terapia cognitivo-comportamental em pacientes tratados com ISRS.

**2. Objetivos**

2.1. Avaliar a eficácia e a segurança de um inibidor seletivo de recaptação da serotonina (ISRS) comparado ao placebo no tratamento de 20 semanas de pacientes com diagnóstico primário de fobia social.

2.2. Avaliar se a adição de terapia comportamental (TCC) à terapêutica farmacológica (manejo clínico convencional) traz benefícios adicionais no tratamento destes pacientes, ao longo de 20 semanas.

2.3. Avaliar a eficácia da Terapia comportamental cognitiva comparada com terapia psicodinâmica em pacientes com diagnóstico primário de fobia social.

2.4. Avaliar o efeito dos tratamentos sobre a aquisição de habilidades sociais em cada um dos grupos.

**3. Métodos**

3.1. Sujeitos

- - 1. Critérios de inclusão

1. 128 pacientes do Ambulatório de Ansiedade (AMBAN) do Instituto de Psiquiatria do HC-FMUSP
2. idade entre 18 e 65 anos
3. diagnóstico de Fobia Social (DSM-IV), de pelo menos um ano de duração feito por um psiquiatra utilizando a Entrevista Clínica Estruturada para o DSM-IV (First et al, 1996)
4. com escores iguais a superiores a 4 na escala de Impressão Clínica Global (CGI; Guy, 1976) e iguais ou superiores a 17 e 24 na SAD e FNE (Watson; Friend, 1969), respectivamente.

3.1.2 Critérios de exclusão

1. diagnóstico de depressão maior com risco de suicídio ou com BDI (Beck, 1961), maior ou igual a 30 ou com pontuação na HAMD (Hamilton, 1960) maior ou igual a 21
2. qualquer outro diagnóstico psiquiátrico primário do DSM-IV, que não o de Fobia Social
3. doença orgânica, epilepsia
4. ingesta superior a 2 unidades de álcool/dia
5. pacientes utilizando medicação antidepressiva e benzodiazepínicos

3.2. Instrumentos:

3.2.1 De avaliação de sintomas ansiosos:

1. HAMA (HAMILTON, 1960)

3.2.2 De avaliação de sintomas depressivos

1. Escala de Depressão de Hamilton (HAMD; HAMILTON, 1959)
2. Inventário de Depressão de Beck (BDI; BECK et al., 1961)

De avaliação da gravidade dos sintomas fóbico-sociais

1. Escala de desconforto e esquiva social (SAD; WATSON; FRIEND, 1969)
2. Escala de medo de avaliação negativa (FNE; WATSON; FRIEND, 1969)

De avaliação do funcionamento global

- Escala de impressão clínica global (CGI; GUY, 1976)

3.3. Planejamento experimental

O estudo será realizado com 4 grupos com 32 pacientes em cada grupo. No grupo I será ministrada droga ativa e Terapia comportamental em grupo, sendo possível, portanto, avaliar a adição da TCC ao ISRS; no grupo II será ministrada a droga ativa com terapia psicodinâmica em grupo, podendo ser avaliado o efeito do ISRS independente da TCC; no grupo III, será ministrado placebo e TCC em grupo, podendo ser avaliado o efeito da TCC e, no grupo IV será ministrado placebo e terapia psicodinâmica em grupo, podendo ser avaliados os efeitos inespecíficos dos tratamentos.

Os grupos terapêuticos serão compostos de 8 pacientes, sendo que cada célula do desenho experimental terá 4 grupos de psicoterapia. Os 128 pacientes serão divididos aleatoriamente por ordem de chegada nestes grupos. Ao mesmo tempo, os pacientes receberão aleatoriamente sertralina ou placebo. O desenho experimental pode ser analisado no quadro l.

# QUADRO 1

# Desenho experimental

Grupo I – 32 sujeitos Grupo III – 32 sujeitos

TCC TCC

Sertralina Placebo

Grupo II- 32 sujeitos Grupo IV – 32 sujeitos

Terapia Psicodinâmica Terapia Psicodinâmica

Sertralina Placebo

3.4 Procedimentos e avaliação

Os pacientes triados no ambulatório de ansiedade (AMBAN) serão encaminhados para uma entrevista diagnóstica e exame físico com um médico psiquiatra. Durante essa entrevista (semana -2) os pacientes serão avaliados para confirmação diagnóstica, preencherão termo de consentimento e serão submetidos a 2 semanas de "wash-out” recebendo uma cápsula de placebo (lactose) por 15 dias.

Em seguida serão encaminhados à avaliação psicológica, que inclui os instrumentos de avaliação de habilidades sociais. Posteriormente os pacientes serão randomizados para um dos 4 grupos de tratamento combinado. O tratamento terá a duração de 20 semanas.

Os pacientes que ao final do período de wash-out com placebo, que obtiver um escore inferior a 17 na SAD e inferior a 24 na FNE serão encaminhados ao ambulatório assistencial didático do AMBAM para tratamento aberto e excluídos do estudo.

Em qualquer momento do estudo em que a pontuação no BDI exceder a 30 pontos e ser superior a 21 pontos na HAMD o caso deverá ser discutido com a equipe, para avaliar cada caso e como proceder em cada um deles.

Sertralina e Placebo serão administradas em cápsulas idênticas. Placebo para a fase de wash-out será administrado em vidros separados rotulados como Frasco 0. Na fase duplo cega do estudo os vidros serão numerados de Frasco 1 a Frasco 20.

As sessões de avaliação e acompanhamento medicamentoso serão realizadas durante as semanas 0, 1, 2, 4, 8, 12, 16 e 20 para os 4 grupos de tratamento, com duração de 45 minutos cada uma.

Uma vez que a dose ótima (dose mais eficaz e tolerável) seja alcançada, o paciente deverá ser mantido com essa dosagem a não ser que apareçam efeitos colaterais que obriguem a uma redução ou retirada da medicação. O médico deverá optar por uma redução inicial à dose anterior antes da suspensão da medicação.

As doses serão padronizadas conforme ilustra a figura abaixo:

|  |  |  |  |  |  |  |  |
| --- | --- | --- | --- | --- | --- | --- | --- |
|  |  |  |  |  | +3 | +3 | +3 |
|  |  |  |  | +2 | +2 | +2 | +2 |
|  |  |  | +1 | +1 | +1 | +1 | +1 |
|  |  | SD | SD | SD | SD | SD | SD |
|  | PRÉ-SD |  |  |  |  |  |  |
| Semana -2 | Semana 0 | Semana 1 | Semana 4 | Semana 8 | Semana 12 | Semana 16 | Semana 20 |
| WASH OUT | VISITA 0 | VISITA 1 | VISITA 2 | VISITA 3 | VISITA 4 | VISITA 5 | VISITA 6 |

SD = Dose padrão definida (ex. 50 miligramas de sertralina). Os pacientes receberão uma dose que irá variar de 1 a 4 vezes esta dose padrão.

+1 = 1^a^ opção de aumento de dose

+2 = 2^a^ opção de aumento de dose

+3 = 3^a^ opção de aumento de dose

Os pacientes alocados nos grupos I e III receberão 20 sessões de 90 minutos, 1 vez por semana, de terapia comportamental cognitiva (TCC) em grupo. Elaborou-se um Manual do Pesquisador para o atendimento dos pacientes para que os terapeutas tenham o mesmo procedimento de atuação. Os pacientes alocados nos grupos II e IV receberão 20 sessões de 90 minutos cada de terapia psicodinâmica em grupo. A terapia de psicodinâmica foi definida como suporte terapêutico em que o profissional não dará instruções de exposição e não realizará treino de habilidades sociais. Será uma ocasião em que o paciente poderá trocar experiências com seu grupo através de depoimentos e em que os fatores inespecíficos da terapia estarão presentes. O roteiro de atuação destes terapeutas foi pré-determinado, sendo elaborado um manual de atuação.

As sessões de terapia serão gravadas. A análise do conteúdo das sessões será feita por 2 avaliadores que ouvirão 30% das fitas, escolhidas aleatoriamente.

3.5. Análise estatística

As hipóteses de nulidade são:

- Não há diferença clínica entre ISRS e placebo no tratamento agudo de fóbicos sociais

- A adição de TCC não traz benefícios ao tratamento com ISRS ao longo de 20 semanas nestes pacientes

- Não há diferença entre TCC e terapia de apoio para o tratamento de fóbicos sociais
- Não há diferença na aquisição de habilidades sociais entre os grupos.

Serão utilizadas na análise, os últimos dados observados (last observation carried forward). Os sujeitos com avaliação na linha de base e pelo menos uma avaliação de acompanhamento serão incluídos na amostra pela intenção de tratar. Serão utilizados os testes de Kolmogorov-Smirnov e de Levene para avaliar a normalidade da distribuição e a homogeneidade das variâncias respectivamente antes das análises estatísticas. Serão utilizados testes-t para examinar diferenças entre os grupos. Testes de Mann-Whitney serão utilizados para examinar variáveis que não tenham distribuição normal. Dados categoriais serão comparados utilizando teste de qui-quadrado e teste exato de Fisher. ANOVA multifatorial com medidas repetidas será utilizada para examinar mudanças ao longo do tempo entre os grupos. As comparações de pares planejadas são as seguintes: sertralina versus placebo; terapia cognitiva comportamental versus terapia psicodinâmica; Grupo I versus Grupo III; Grupo II versus Grupo IV. O valor de alfa foi definido como 0,05 para significância. Quando necessário, o alfa será ajustado para múltiplas comparações.

**3.6. Ética**

O projeto está de acordo com a resolução N^o^ 196 do CONSELHO NACIONAL DE SAÚDE, de 10 de outubro de 1996.

Todos os pacientes deverão ter pleno conhecimento dos objetivos e métodos do experimento e deverão dar seu consentimento por escrito.

**4. Referências bibliográficas**

AL-KUBAISY, T.; MARKS, I.M.; LOOSDAIL, S. Role of exposure homework

in phobia reduction: a controlled study. Behavior Therapy, v.23, p.599-621, 1992.

ALNAES, R.; TORGERSEN, S. The relationship between DSM-III

symptom disorders (Axis I) and personality disorders (axis II) in an

outpatient population. **Acta Psychiatr. Scand.,** v.78, p.485-92, 1988.

ALSTROM, J.E.; NORDLUND, C.L.; PERSSON, G. The effects of four

treatment methods on social phobic patients not suitable for insigth-oriented psychoterapy. **Acta Psychiatrica Scandinavica,** v.70, p.97-110, 1984.

AMERICAN PSYCHIATRIC ASSOCIATION. **Diagnostic and**

**statistical manual of mental disorders**, 3. ed. Washington, DC, American Psychiatric Association, 1980.

AMERICAN PSYCHIATRIC ASSOCIATION. **Diagnostic and**

**statistical manual of mental disorders**, 3. ed. rev. Washington, DC, American Psychiatric Association, 1987.

AMERICAN PSYCHIATRIC ASSOCIATION. **Diagnostic and**

**statistical manual of mental disorders,** 4. ed. Washington, DC, American Psychiatric Association, 1994.

BARLOW, D.H. **Anxiety and its disorders**. New York, Guilford, 1988.

CABALLO VE. Manual de Evaluación y Entrenamiento de las Habilidades Sociales. Spain: Siglo XXI de España; 1993

COX, B.J.; DIRENFELD, D.M.; SWINSON, R.P.; NORTON, G.R.

Suicidal ideation and suicidal attempts in panic disorders and social phobia. **Am. J. Psychiatry,** v.151, p.882-7, 1994.

DAVIDSON, J.R., POTTS, N., RICHICHI, E., KRISHNAN, R., FORD, S.M.,

SMITH, R., WILSON, H.T. Treatment of social phobia with clonazepan and placebo. **J. Clin. Psychopharmacology,** v.13, p.423-8, 1993.

FIRST MB, SPITZER, ROBERT L, GIBBON MIRIAM, AND WILLIAMS, JANET B.W. Structured Clinical Interview for DSM-IV Axis I Disorders, Clinician Version (SCID-CV). . Washington, D.C.: **American Psychiatric Press, Inc**; 1996.

GUY, W ECDEU Assessment manual for psychopharmacology, revised NIMH,

Publishers, Bethesda, 1985

FAHLEN, T. Personality traits in social phobia. II: Changes during

treatment. **J. Clin. Psychiatry,** v.56, p.569-73, 1995.

HAMILTON, M. The assessment of anxiety states by rating. **British Journal of**

**Medical Psychology**, v.32, p.50-55, 1959.

HAMILTON, M A rating scale for depression. **J. Neurol Neurosurg Psychiatry**, v.

23, p. 56-62, 1960.

KATZELNICK, D.J.; KOBAK, K.A.; GREIST, J.H.; JEFFERSON, J.W.;

MANTLE, J.M.; SERLIN, R.C. Sertraline in social phobia: a double-blind placebo-controlled crossover study. **Am. J. Psychiatry,** v.152, p.1368-71, 1995.

LEPOLA, U.; KOPONEN, H.; LELNONEN, E. Citalopran in the treatment of

social phobia: a report of three cases. **Pharmacopsychiatry.** v.27, p.186-8, 1994.

LOTUFO-NETO, F.; GENTIL, V. Alcoholism and phobic anxiety- a

clinical-demographic comparison. **Addiction,** v.89, p.447-53, 1994.

MANCINI, C.; VAN AMERINGEN, M. V. Paroxetine in social phobia. **J.Clin. Psychiatry.** v.57, p.519-22, 1996.

SUSSMAN, N. Treating anxiety while minimizing abuse and

dependence. **J. Clin. Psychiatry,** v.54, p.44-51, 1993. Supplement 5.

TURNER, S.M.; BEIDEL, D.C.; JACOB, R. Social phobia: a comparison of

behavior therapy and atenolol. **J.Consult. Clin. Psychol.,** v.62, p.350-358, 1994.

VAN AMERINGEN, M.; MANCINI, C.; STYAN, G.; DONISON, D.

Relationship of social phobia with other psychiatrc illness. **J. Affect. Disord.,** v.21, p.93-9, 1991.

VAN AMERINGEN, M.; MANCINI, C.; STREINER, D. L.; Sertraline in social

phobia. **J. Affect Dis.,** v.31, p.141-5, 1994.

VAN VLIET, I.M.; DEN BOEF, J.A.; WESTENBERG, H.G.;

Psychopharmacological treatment of social phobia; a double blind placebo controled study with fluvoxamine. **Psychopharmacology,** v.115, p.128-34, 1994

VERSIANI, M.; NARDI, A.E.; MUNDIM, F.D.; ALVES, A.D.;

LIEBOWITZ, M.R.; AMREIN, R. Pharmacoterapy of social phobia. A controlled study with moclobemide and phenelzine. **Br. J. Psychiatry,** v.161, p.353-60, 1992.

WATSON, D.; FRIEND, R. Measurement of social-evaluation anxiety. **J. Consult**.

**Clin. Psychol**., v. 33, p. 448-457, 1969

WLASLO, Z.; SCHOEREDER-HARTWIG, K.; HAND, I. Exposure in vivo

versus social skills training for social phobia: Long-term outcome and differential effects. **Behav. Res. Therapy,** v.28, p.181-193, 1990.

WORLD HEALTH ORGANIZATION. **Mental disorders: glossary**

**and guide to their classification in accordance with the 9th revision of I.C.D.**  Geneva, World Health Organization, 1978.

WORLD HEALTH ORGANIZATION. **International classifications of**

**diseases.** 10. rev. Geneva, World Health Organization, 1989.
